# Female Monarch Butterflies Are More Successful than Males During the Fall Migration: A Literature Review

**DOI:** 10.3390/insects17101056

**Published:** 2026-10-13

**Authors:** Andrew K. Davis

**Affiliations:** Department of Ecology, Evolution, and Organismal Biology, Kennesaw State University, Kennesaw, GA 30144, USA; adavi749@kennesaw.edu

**Keywords:** insect migration, monarch butterflies, *Danaus plexippus*, energy, aerodynamics, melanism, sex differences

## Abstract

The migration of monarch butterflies in North America is one that has been shaped by thousands of years of adaptation. Over the last century, studies of monarch migration have often reported subtle differences in migration-related traits between male and female monarchs. This literature review aimed to organize these disparate reports, which together provide evidence that female monarchs are superior to males in their migration success. Females have higher tag-recovery rates from Mexico, they have greater lipid stores during the migratory journey, and they arrive in Mexico with more fat than males. They also show lower instances of wing damage, both during migration and at arrival to the winter colonies, indicating their migration is less physically taxing. The mechanism for this is not physiological, as there are no sex differences in metabolism during flight, nor are there navigational differences. It is likely a matter of aerodynamics and flight efficiency; females have lower whole-body wing loading indices and their greater wing melanism levels may also be a factor. Female wings are ~58% black-pigmented, compared to ~48% on males. This basic biological difference has never been explained. Studies from other winged animals show how black pigment absorbs solar energy, which can heat the upper wing surface and reduce drag. Since monarchs have a pronounced dimorphism in melanism, but yet other non-migratory Danaid butterflies do not, it can be argued that the monarchs’ unique migration and overwintering phase has driven this greater female melanism. Females would need to retain more lipids over the winter months to have enough left over to develop eggs in the spring. From an engineering standpoint, this sex difference in flight ability could be an avenue for research into the mechanics of flight, i.e., bioinspiration.

## Simple Summary

Over the last century, studies have often reported subtle differences in migration-related traits between male and female monarchs. Here, I organize these disparate reports, which together provide evidence that female monarchs are superior to males in their migration ability. Females have higher tag-recovery rates from Mexico, they have greater lipid stores during the migratory journey, and they arrive in Mexico with more fat than males. They show lower instances of wing damage than males, indicating their migration is less physically taxing. Meanwhile, there are no sex differences in metabolism during flight, nor in navigational ability. Females have lower whole-body wing loading indices, which may explain some of the efficiency, though their greater wing melanism levels may also be a factor. The fact that female monarch wings are more darkly pigmented has never been explained. Studies from other winged animals show how black pigment absorbs solar energy (especially during soaring flight), which can heat the upper wing surface and reduce drag. Possible reasons for these findings are discussed, including the possibility that females would need to retain more lipids over the winter months to have enough left over to develop eggs in the spring.

## 1. Introduction

**Figure 1:**
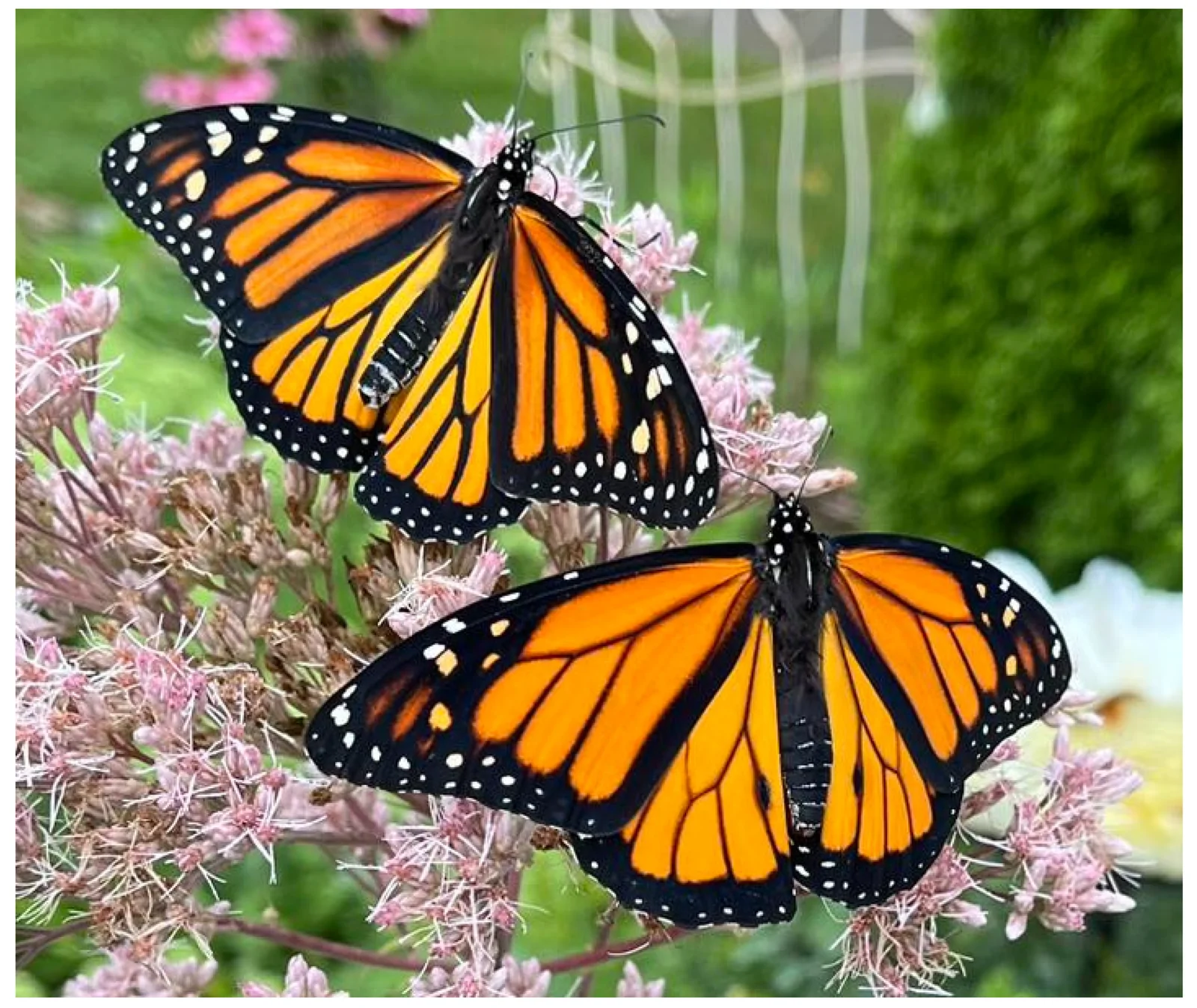
Picture of female (**top**) and male (**bottom**) monarch butterflies. Photo taken by Jennifer Marsh O’Reilly.

Each fall, monarch butterflies (*Danaus plexippus*, Figure 1) in eastern North America attempt a 3000 km migration that lasts as much as 2 months to complete, ending at wintering colonies located in mountainous regions of Central Mexico. After the winter period, the surviving adults engage in reproduction while returning northward to recolonize the breeding range. This journey has fascinated scientists for decades, ever since the locations of the wintering colonies were discovered in 1975 [1]. However, even before the discovery, researchers had often studied southward-flying monarchs in the fall, noting the behaviors [2], physiology [3,4,5], and anatomical traits [6] that allow these insects to fly so efficiently. Despite this wealth of knowledge on this phase of the annual cycle, certain basic questions still remain about the migration, including even whether male and female monarchs migrate equally well, which is the topic of this review. In fact, there have been indications in recent years that they may not; specifically, analyses of tag-recovery rates from two different studies showed that females tend to have higher recovery rates in Mexico [7,8]. This trend provided the launching point for the current literature review, where the author set out to discover what other migration-related traits could be different between male and female monarchs, including how much lipids they accumulate during the journey and whether they incur wing damage in equal rates, among other important measures of “migration success.” The results of this literature review revealed additional evidence showing the superiority of female monarchs during the fall journey, which in itself raises intriguing questions about their morphological differences and whether this contributes to their migration success. In fact, the very fact that female monarchs are more darkly pigmented than males (Figure 1) has never been explored or explained by science, and it may be a key factor in the females’ migration success. The sections below describe the results of this literature review and are organized by topic, according to the specific migration traits in question. A brief summary of the published findings from all topics below is presented in Table 1.

## 2. Fat Reserves

The monarchs’ fall migration is fueled by energy in the form of fat reserves, which they accumulate during the journey [4,9,45,46,47]. Some energy reserves are accumulated during the larval stage, which carry over into the newly transformed adult, and so first I describe the available evidence from this life stage. One early study conducted lipid assays of newly eclosed males and female monarchs [13], and reported that females tended to have less lipid reserves than males. Another early study of newly eclosed monarchs found mixed results comparing male and female lipid levels [14]. Similarly, a later study that indirectly examined energy reserves of newly eclosed monarchs using residual mass [15] also found that females tended to have lower reserves than males at the beginning of the adult stage.

Regarding the energy acquired during the migration, if either sex accumulates or retains more lipids during the journey, or especially, upon arrival to wintering grounds, it would indicate either greater success at finding nectar sources, greater energy efficiency during flight itself, or perhaps less overall energy demand across the entire journey. In at least four different studies, female monarchs sampled during the fall migration were found to have more lipids (proportional to their body size) than males. This was shown in Ontario [3], Virginia [9], Missouri [5], Florida and Cuba [10]. One study from Georgia found no sex differences in fat loads of migrants [11]. Also, two studies showed that females arrive at the Mexico winter colonies with greater fat reserves [12,47], and one study showed the same pattern at the California winter colonies [14]. Collectively, these studies (mostly) point to the same pattern, that female monarchs are able to complete the migration with greater fat reserves. The fact that this consistency across studies was never noticed may relate to the way the data were reported in each case, which was by simply reporting the mass of lipids for each sex, and which do not appear visually different. However, the generally smaller size of female monarchs [24,48] means that, proportionally, female monarchs do indeed have greater fat reserves than males, according to these studies.

Another way to evaluate differences in fat loads is to look at studies where monarch “body condition” was reported, which is an index of size-adjusted body mass. This measure does not require dissections or lab-based assays, only a measure of body size (typically wing length) and a measure of body mass, and then the body condition index can be calculated. Note that this index is crude, especially since the “body size” index is wing length, not the size of the actual butterfly body. This literature review identified three such studies of monarch migrants, and in all cases, females had better body condition scores than males; this was reported from indices in Virginia [8], Minnesota [15] and Texas [16]. Put another way, females had greater mass, given their body size, than males, which likely comes from greater fat reserves. Thus, while indirect, this evidence is consistent with the studies where actual fat loads were measured. Together, this is a significant piece of evidence that in itself speaks volumes about the overall differences in migration success between male and female monarchs; females appear to be better able to save energy (lipid reserves) than males over the same extent of travel.

## 3. Tag-Recapture Rate During Migration Stopover (Stopover Length)

Migrating monarchs must stop periodically during the long-distance journey to rest and/or to forage for nectar, which is converted into fat reserves [9]. As such, it would be of interest to know if there are any sex differences in this behavior, such as differences in the length of time spent at these “stopover” sites. If so, this might explain the greater fat loads of females during the journey (perhaps they remain longer to forage during their stops). As far as the author is aware, this (stopover lengths) has only been studied twice. In one study of stopover lengths in coastal Virginia (using only data from one fall season), Davis and Garland [18] reported no sex-related differences in stopover length, based on mark-recapture efforts. However, examination of stopover lengths from a multi-year effort in South Carolina, McCord and Davis [17] indicated a clear sex-based difference; females tended to have shorter stopover durations than males. While this evidence goes against the initial thought above, it could still be interpreted as support for the findings above concerning fat loads, in that female monarchs require less stopover time during the migration (for refueling). However, considering the lack of consistency between studies, and the dearth of research on this topic, further speculation is not warranted.

## 4. Wing Shape and Pigmentation

Wing shape is important for animals that migrate long distances, as longer, pointier, wings are thought to be more aerodynamic [49,50,51]. Multiple studies have examined the shape of monarch forewings (which extends laterally during soaring flight) and measured males and females separately, though the findings concerning sex differences are varied. Altizer and Davis [24] found no sex differences in forewing aspect ratio (ratio of length to width), as did Tenger-Trolander et al. [26] and Soule et al. [21], but Tenger-Trolander et al. [27] and Li et al. [28] did find that females had a greater aspect ratio (more elongated forewings) when measured using more precise shape estimation. Meanwhile, Freedman and Dingle [52] found that males had slightly more elongated forewings than females. Finally, Dockx [30] found migrant females in Texas had more elongated forewings than males. Considering all evidence above, there appears to be minimal support for female monarchs having more elongated forewings, which if true, would theoretically make their flight more aerodynamically efficient.

Aside from wing shape, it is also important to consider the monarchs’ basic biological difference in wing pigmentation in the conversation about migration success, as studies of other flying taxa are now revealing how black pigmentation in particular has important aerodynamic impacts, because of its solar energy absorption capacity [53,54,55]. While it has long been known that female monarchs have greater amounts of black pigment on their wings than males (see Figure 1), modern image analysis approaches have now allowed this difference to be quantified; Johnson et al. [56] showed that males have 42–52% of their wings covered in black, compared to 54–62% black in females. The functionality of this greater wing melanism has never been fully studied, though there is some evidence that it does convey greater migration success in monarchs; a study of spring-migrating monarchs showed that greater wing melanism was correlated with migration distance [57]. Surprisingly, then, a more recent study of monarchs during the fall migration showed no evidence for this melanism effect [58], but instead, found that the size of the outer white spots of monarch wings was the better predictor of migration success. The explanation was thought to be that the combination of white spots, arranged along the outer edge where the margin is black, creates micro-regions of heating and cooling, which then reduce air drag along the upper wing surface. With this in mind, it is important to consider that female monarchs do have larger white spots than males [58], which means their drag resistance may be lower than males. This is an issue that is deserving of further study because of its relevance to the engineering field of “bioinspiration”.

## 5. Wing Strength and Damage-Resistance

Wing damage can greatly impact the flight performance of any flying insect [59,60,61], so this trait (damage resistance) would be of obvious importance to migrating monarchs. This literature review found that female monarchs consistently show less wing damage during migration. Migrant females had less wing damage than males in Texas [16], females had better wing condition scores (a composite of damage and wear) in South Carolina [31], and females had less damage than males when they arrived at California winter colonies [32]. Interestingly, this pattern appears not related to differences in wing tensile strength, which was once tested using a force gauge, and found to be similar in male and female monarchs [25]. Combined, this evidence therefore suggests that the journey of female monarchs is somehow less physically taxing than that of males. Either females are engaging in less damaging behavior during the journey (perhaps while roosting?) or the flight itself is less demanding to female wings.

Missing from this line of inquiry are any studies that compare actual in-flight differences (or similarities) in actual flight behavior. For example, one explanation for the wing damage differences is that females engage in more soaring flight than flapping, which may reduce the wear and tear over the two-month journey. In the author’s thorough literature search, no such studies were found.

Another possible explanation for the wing damage pattern is that the more darkly pigmented female wings actually provide increased structural support. This idea is supported by the fact that female monarch wings are actually heavier, per unit area, than those of males [23]. Moreover, there is evidence from other taxa that melanin pigment does provide strength to tissues [62]. However, since the actual black pigment of monarchs is on their wing scales (not the wing tissue), it is hard to imagine how scales themselves provide physical support. Thus, while the finding here of sex-differences in wing damage “resistance” is in-line with the prior evidence reported, the mechanism behind it awaits further study.

## 6. Flight Mill Evidence

One indirect way to gauge how well monarchs can migrate is to actually test their flight ability, which can be tested using constructed flight mills, in which a monarch is glued to a rod that is attached to a central pivot, and the butterfly then “flies” in circles while a computer tracks its speed and distance. While this approach only allows monarchs to flap (i.e., not soar), it does provide information on the factors that influence important flight metrics [21,33,63]. Of the various studies that have reported using these devices, some have reported sex-related differences, but not all. Two studies showed that female monarchs tended to fly longer (i.e., farther) than males [19,20], but Soule et al. [21] reported no sex differences in flight time. In terms of flight speed, Bradley and Altizer [20] reported no sex differences, but in an unpublished Master’s thesis, Ballew [22] showed that females, but not males, increased their flight speed when fed high-quality nectar. Collectively, there appears to be weak support for the idea that females have better “flight ability” than males, using these lab-based devices.

## 7. Navigation and Orientation

The greater tag-recovery rates of females over males at the wintering colonies could simply stem from female monarchs being better at finding their way during the journey (i.e., navigation). There have been multiple studies published where monarch “navigation” or “orientation” ability has been tested and where researchers made note of the sexes in their tests. In all cases, no sex-related differences were noted. In an unpublished Master’s thesis, Aikins [37] reported that groups of wild-caught, migrant female and male monarchs oriented to the same side when held in an outdoor tent. In flight simulators that tracked individual directions, neither Zhu et al. [64] nor Wilcox et al. [65] reported sex-related differences in orientation of fall migrants. The lack of sex differences in all orientation trials argues that females are not better at navigating during the fall migration. This evidence is also consistent with lab-based, cognitive tests, where male and female monarchs were reported to have the same ability to learn and/or remember [40].

## 8. Migration Distance Inferred from Isotopes

An alternative approach to delineating migration pathways of migratory animals is to examine tissues of collected specimens for isotopic signatures of hydrogen and carbon, which can inform as to where in North America the specimen was born [66]. This approach has proven valuable for determining which region of North America wintering monarchs had come from [67,68], or, where actively migrating monarchs originated [69]. Importantly, two studies have separately examined isotopes of (wintering) male and female monarchs to gauge their migration distances. Both studies reported similar findings; no sex differences in inferred migration distance were found by Altizer et al. [35], or Flockhart et al. [67]. A related study of migration distances of western monarchs did not separately report male and female distances inferred from isotope data [48]. The overall lack of evidence on this topic may or may not be important for this literature review, as it could simply be interpreted as showing that both sexes travel across the same migratory routes. On the other hand, the lack of differences found in these studies may stem from the coarseness of the approach for “tracking” specimens; examining isotopes only allows for assigning origins to broad geographic regions in North America.

## 9. Metabolic Rate

This author considered that the superior flying efficiency of female monarchs could be because of inherent sex-related differences in metabolism, where females may require less energy expenditure for locomotion, perhaps because of their (generally) smaller body size [24] or their lower wing loading [23]. However, this is not the case; in a lab-based investigation into resting metabolic rates, Tenger-Trolander et al. [26] reported no differences between summer-reared, or fall-reared, males and females. Also, two lab-based studies that examined metabolism of monarchs either “in flight,” or “post-flight” also reported no notable sex-related differences in oxygen consumption rates [33,34]. Importantly, the rates in all cases were adjusted for body size differences. The consistency within these studies argues that each sex uses the same amount of energy for basic bodily functions, and importantly, for flight.

## 10. Natural and Anthropogenic Mortality Risks

The two-month journey to central Mexico requires overcoming numerous natural and unnatural obstacles and/or stressors, and so it is of interest to know if females show greater survival in these cases, which may explain their better winter recovery rates. One set of rather dire manmade obstacles is the thousands of miles of highways and roads along the migration flyway, which result in “millions” of monarch deaths each year [41,42,70]. Of the roadway survey studies which reported male and female deaths separately, McKenna et al. [41] found 36% of roadkill monarchs in Illinois were females, and Kantola et al. [42] found 38% were female in Texas. On one hand, this could be interpreted as evidence that females are less likely to be killed on roadways, though there is also the possibility that these ratios reflect male-biased sex ratios during the fall [7,71]. The same problem arises when considering any sex-related differences in mortality stemming from crossing natural water bodies, or suffered during storms. In a very early study, Beall [43] reported a collection of 33 dead monarchs washed up on the shore of Lake Erie, of which 11 (33%) were female. In a later study, Howard and Davis [44] reported on a similar mortality event on the shore of Lake Michigan, of which 45% were female. Again, these could be evidence of lower mortality risk by females, or, simply a male-biased sex ratio during the fall. Interestingly, this sex ratio pattern seen in the fall is not related to sex differences in “catchability” [31,72].

## 11. Synthesis

This literature review has revealed a surprising biological pattern that appears to have gone unnoticed throughout the many years where monarchs have been studied: female monarchs are more successful in their fall migration than males. The most important pieces of evidence appear to be that (1) tagged females are more often found at their winter destination (direct tag recoveries); (2) they accumulate and arrive at the colonies with greater fat reserves; and (3) they incur less damage during the trip. This success appears NOT related to superior navigational ability, cognitive ability, or differences in metabolism or energy consumption, but is likely more related to superior aerodynamic efficiency. Specifically, despite being slightly smaller than males, females have lower whole-body wing loading (reduced cost of movement), and they have greater melanization, which in other species is known to allow for greater solar warming of the wing surface, which in turn reduces drag. Finally, their outer white spots are also bigger, which could provide extra drag-reduction [58].

An alternative explanation for the above findings is that female monarchs may simply fly higher than males during the migration, which would allow them to save energy through soaring and incur less injuries from ground-level mishaps. Indeed, there is lab-based evidence that monarch flight efficiency is theoretically more efficient at higher altitudes [70]. However, to the author’s knowledge, there have been no studies separately comparing flight altitudes of the sexes. This is despite extensive research and observations of flying monarchs [10,71,72]. Unfortunately, males and females have never been examined separately in such studies, perhaps owing to the difficulty in discerning them from a distance. This potential explanation therefore awaits further study.

This discovery in turn raises the obvious question: why would female monarchs need to be better at migrating in the first place? Females take the same migration routes as males do, navigate through the same gauntlet of obstacles, deal with the same water crossings, road crossings, cross drought-stricken landscapes, etc., yet apparently, are more successful than males, both at simply reaching the destination, but also doing so with more accumulated fat reserves and less physical damage. One possibility is that females would need to have greater fat stores during the winter to be able to produce eggs and/or re-migrate northward in the spring, so as to begin the next generation. Thus, their enhanced fall migration success could be an adaptation to ensure that they arrive to the winter colonies with sufficient fat reserves to not only survive the winter months, but to then re-migrate northward and repopulate the spring generation (i.e., produce eggs). Consider that the migratory generation has delayed development of their reproductive tissues (i.e., reproductive diapause) until the spring [73]. Egg production in Lepidoptera does require a heavy investment of lipids [74]. Since monarchs do not acquire lipids during the winter months [9,46,75], all of this energy requirement in the spring must come from the females’ stored lipids that were acquired during the previous fall migration [47,76]. Thus, the higher energetic needs of female monarchs during this critical phase of the annual cycle could explain their enhanced migration success, and even the sex differences in wing morphology in this species, as explained below.

Female monarchs have darker wings than males, yet to date, this basic biological difference has never been explained, despite centuries of study of this species. Could this morphological difference be the key to the greater migration success of females, and thus explain the sex difference? If this were the case, it would imply that females have evolved greater wing melanism for a biological “need,” as in, a need to be more successful at migrating.

**Figure 2:**
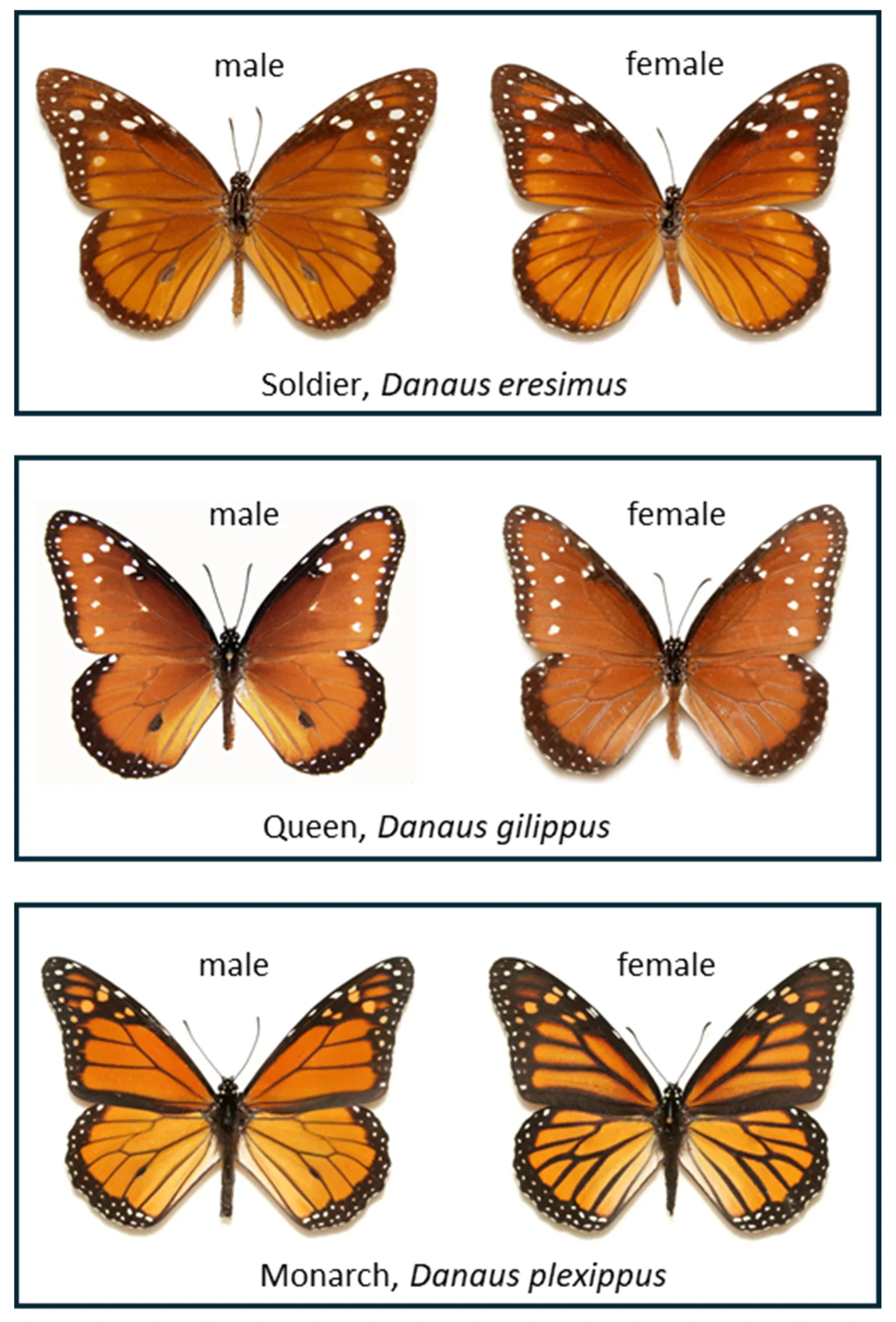
Photographs of pinned specimens of non-migratory soldier (*D. eresimus*) and queen (*D. gilippus*) butterflies, shown for comparison with migratory monarchs (*D. plexippus*). All images courtesy of www.butterfliesofamerica.com (accessed on 6 August 2026).

If this were the case, comparisons with other related, but non-migratory, Danaid butterflies should be of interest, since such species, with no dormant overwintering phase, would not have evolved sex-related differences in melanism. In fact, non-migratory queen butterflies, *Danaus gilippus*, show no visible evidence of enhanced melanism in females compared to males (Figure 2), nor do soldier butterflies, *Danaus eresimus* (Figure 2). This ancillary evidence supports the explanation above regarding the function of the enhanced melanism on female monarchs. Alternatively, the enhanced melanization of female monarchs could reflect a need for enhanced solar heat absorption for egg synthesis (i.e., during the cool spring), of which there is evidence in other species [76]. However, if this were the case, one would expect the same phenomenon in the related *Danaus* species, yet there is none.

Finally, the greater migration success of female monarchs could prove to be valuable in the field of “bioinspiration” and aerospace engineering, whereby researchers learn from nature to improve humanmade machinery or vehicles. Indeed, the incredible flight ability of monarchs has not gone unnoticed, particularly by aerospace engineers [77,78]. In this case, the comparative study of male and female monarch wings and/or flight mechanics could offer clues into how to build more efficient (slow-moving) flying vehicles, such as fixed-wing drones, or other soaring crafts. An obvious place to start these investigations would be to explore how the greater melanism levels of females improve their flight aerodynamics. Adding to this, Davis et al. [58] also showed how the spot sizes are greatly reduced in related species that do not migrate, which argues that large white spots are correlated with migration success. And, while females have greater black coverage on their wings, they also have larger white spots than males. This pattern also deserves more study, because of its relevance to the engineering field. Obviously, there is still much to be uncovered regarding the functional significance of monarch wing patterns, and how exactly they influence flight.

## Figures and Tables

**Table 1:** Summary of studies where male and female monarchs have been studied and the resulting findings.

Measured Trait	Finding and Source(s)
Tag-recovery rate in Mexico	Females had higher recovery rates in studies from Virginia and Pennsylvania [7,8].
Lipid mass during migration	Females had proportionally more in Virginia [9], Missouri [5], Florida and Cuba [10]. Beall [3] also found that migrant females in Ontario had proportionally greater fat loads. No sex differences were found in migrants in Georgia [11].
Lipid mass upon arrival to wintering colonies	Females had more in Mexico colonies [12,13] and in California colonies [14].
Body condition index during migration (mass per unit size)	Females had better indices in Virginia [8], Minnesota [15] and Texas [16].
Tag-recapture rate during migration stopover	Females had lower recapture rates in South Carolina (less likely to remain) [17], but no sex differences were found in Virginia [18].
Flight duration on a flight mill	Davis et al. [19] and Bradley and Altizer [20] found that females tend to fly for longer, but Soule et al. [21] found no sex differences.
Flight speed on a flight mill	No sex differences found by Bradley and Altizer [20], but Ballew [22] showed that females, but not males, increase speed when fed high-quality nectar.
Whole-body wing loading	Females had reduced wing loading indices, measured in different ways [23,24].
Individual wing thickness/heaviness	Females had thicker/heavier individual wings [23,25].
Wing shape (aspect ratio)	Altizer and Davis [24] found no difference in aspect ratio, as did Tenger-Trolander et al. [26] and Soule et al. [21], but Tenger-Trolander et al. [27] and Li et al. [28] found that females had greater AR when measured using more precise shape estimation. Freedman and Dingle [29] found that males had slightly more elongated forewings. Dockx [30] found that migrant females in Texas had a more elongated wing shape.
Wing tensile strength	No sex differences found [25].
Wing damage during migration	Migrant females showed less damage than males in Texas [16].
Wing condition score during migration (wear plus damage)	Females found to have better wing condition in South Carolina [31].
Wing damage upon arrival to winter colonies	Females showed less damage than males in California colonies during Oct, Nov. and Dec. [32].
Flight metabolic rate	No sex differences found by Schroeder et al. [33] or Pocius et al. [34].
Resting metabolic rate	No sex differences found [26].
Migration distance traveled inferred from isotopes	No sex differences found by Altizer et al. [35] or Flockhart et al. [36].
Orientation ability	No sex difference found when wild monarchs were tested in an outdoor cage [37], and no differences found when tested in flight simulators [38,39].
Learning ability of migrants	No sex differences found [40].
Mortality at roadways	McKenna et al. [41] found 36% of roadkill monarchs in Illinois were females. Kantola et al. [42] found 38% were female in Texas.
Mortality at water crossing/storms	Beall [43] reported a collection of 33 dead monarchs washed up on the shore of Lake Erie, of which 11 (33%) were female. Howard and Davis [44] reported on a similar mortality event on the shore of Lake Michigan, of which 45% were female.

## Data Availability

No new data were created or analyzed in this study. Data sharing is not applicable to this article.
